# Ontogeny of the extrafloral nectaries of *Vigna adenantha* (Leguminosae, Phaseolae) and its relation with floral development

**DOI:** 10.1186/s40529-014-0074-2

**Published:** 2014-12-29

**Authors:** Fabiana Soledad Ojeda, Patricia Susana Hoc, Beatriz Gloria Galati, Maria Teresa Amela García

**Affiliations:** 1 PROPLAME-PRHIDEB (CONICET), DBBE, FCEyN, UBA, Buenos Aires, Argentina; 2 Cátedra de Botánica, FAUBA, UBA, Buenos Aires, Argentina

**Keywords:** Extrafloral nectaries, Inflorescences, Ontogeny, Morphology, Anatomy, Ultrastructure, Vigna, Leguminosae

## Abstract

**Background:**

The inflorescences of the genus *Vigna* Savi have extrafloral nectaries (EFNs) among the flowers whose origin is still unknown. The disposition, anatomy and morphology, as well as the ontogeny of the extrafloral nectaries (EFNs) associated with the inflorescences of *Vigna adenantha* (G.F.W. Meyer) Maréchal, Mascherpa & Stainier (Leguminosae, Papilionoideae, Phaseolae) were studied. Besides, the ultrastructure of the secretory stage was described.

**Results:**

The inflorescence, a raceme, bears a brief globose secondary axis in each node with 2 flowers and 5–7 EFNs, which develop in acropetal direction. Each EFN originates from the abscission of a flower bud that interrupts its development, resulting in an elevated EFN. This secretory structure is formed by a ring of epidermal and parenchymatic cells surrounding a group of elongated central cells. The nectary is irrigated by phloem and xylem. Four developmental stages proceed; each one relates to a different embryological stage of the flowers in each secondary axis.

**Conclusions:**

The first functional EFN of each secondary axis of the inflorescence reaches its maturity when both the pollen grains and the embryo sacs are completely developed and the flowers begin to open. The secretion is granulocrine. The following EFNs develop in the same way.

**Electronic supplementary material:**

The online version of this article (doi:10.1186/s40529-014-0074-2) contains supplementary material, which is available to authorized users.

## Background

In the genus *Vigna* Savi, the inflorescences are double racemes whose nodes bear a brief secondary globose axis in which commonly two flowers and one or more extrafloral nectaries (EFNs) develop. These glands have taxonomic relevance as they allow to distinguish *Vigna* from *Phaseolus* L. (McKey [1989]).

Studies about the EFNs of different families of Angiosperms involve morphology (González and Ocantos [2006]; Machado et al. [2008]; Melo et al. [2010]), anatomy (Francino et al. [2006]; Lattar et al. [2009]; Melo et al. [2010]), ultrastructure (Fahn [1987]; Durkee et al. [1999]) and ontogeny (Maheshwari [1954]; Ojehomon [1968]; Leitâo et al. [2002]; Sousa Paiva and Rodrigues Machado [2006]), but some aspects, such as the vascular supply, have not received much attention (Ojehomon [1968]; Nepi [2007]).

The EFNs associated to inflorescences are supposed to originate from aborted floral primordia in Leguminosae (Tucker [2003]) and specially in Papilionoideae (Ojehomon [1968]). In some of them, not only when flower buds abort but also when flowers are shed (Díaz-Castelazo et al. [2005]).

Ojehomon ([1968]) studied the ontogeny of the EFNs of *Vigna unguiculata* (L.) G. W. Walpers; later, Kuo and Pate ([1985]) analysed the anatomy during the secretory period. There are no other reports on the development of the EFNs in *Vigna*.

Ontogenetic studies concerning *V. adenantha* (G.F. W. Meyer) Maréchal, Mascherpa & Stainier only examined the ovule and pollen grain development, in the context of a comparative analysis of the *Vigna-Phaseolus-Macroptilium* (Benth.) Urb. complex (Faigón Soverna [2002]; Faigón Soverna et al. [2003]).

The aim of this work was to find out the origin and development of the EFNs, the cytology of the secretory stage, as well as the period of secretion and its relation with fruit and seed production in *V. adenantha.* Besides, the ontogeny of the first EFN on each secondary axis of the inflorescence was correlated with the pollen and embryo-sac development.

## Methods

The studied material was collected from cultivated specimens at the Campo Experimental of the Facultad de Ciencias Exactas y Naturales (Universidad de Buenos Aires) situated in the Ciudad Autónoma de Buenos Aires, Argentina.

The cultivated specimens proceeded from: ARGENTINA. Prov. Corrientes: Dpto. Capital, E.B.C.O., 20/02/10, P. S. Hoc 396 (BAFC). Prov. Entre Ríos: Dpto. Concordia, Parque San Carlos, 23/03/02, P. S. Hoc 377 (BAFC); 27/02/10 P. S. Hoc 397 (BAFC). Prov. Buenos Aires: Pdo. Zárate, Puente Zárate Brazo Largo, 23/03/02, Hoc 378 (BAFC).

For observations with optical microscopy (OM) the inflorescences were fixed in FAA (formaldehide, ethanol, acetic acid, water) and preserved in ethanol 70%. Each node, from the apex to the base of the inflorescence, was sectioned, identified with a code, embedded in paraffin and cutted in sections 10 μm thick employing a microtome (Arcano). Histological slides were prepared: some of them were stained with safranin-fast green and others with cresyl violet. Observations and photographs were performed with an optic Nikon Labophot microscope.

Preparations for scanning electronic microscopy (SEM) were performed in the following way: each secondary axis was dehydrated in an ascendant series of alcohols (70, 80, 90, and 100%), submitted to critical point, covered with a gold-palladium alloy and observed and photographed with a Zeiss Supra 40 Scanning Electron Microscope.

In addition, the secretory stage was examined with transmission electron microscopy (TEM). For this, the material was fixed in glutaraldehyde 2.5%, soon after it was submerged in buffer phosphate during 24 hours, then fixed in osmium tetroxide (OsO_4_) 1.5% at 2°C for 3 hours, dehydrated in an upward series of acetone and embedded in Spurr’s resin. For previous observations with light microscopy, sections of 1 μm thickness were stained with toluidine blue 0.1%. Fine sections were stained with uranil acetate and lead citrate, observed and photographed with a Jeol-Jem 1200 EXII transmission electron microscope.

Nectar concentration was measured in a natural population in Concordia (Entre Ríos province, Argentina) as follows: to exclude ants from the EFNs, a thin stripe of aluminium paper was wrapped around the peduncle and each stripe was impregnated with an ant deterrent resin (Tanglefoot, USA). Nectar accumulated in several nectaries was removed with a 1 μl capillary tube. The column of collected nectar was measured in the capillary and the volume calculated. Nectar concentration was registered with a hand refractometer for small volumes (Bellingham & Stanley, UK). In cases in which volume was to small to get a measurable reading, a known volume of distilled water was added to the refractometer and the corresponding calculation was made, taking into account the dilution factor.

Photograhic plates were compounded with Adobe Photoshop software.

## Results

### General morphology

**Figure 1:**
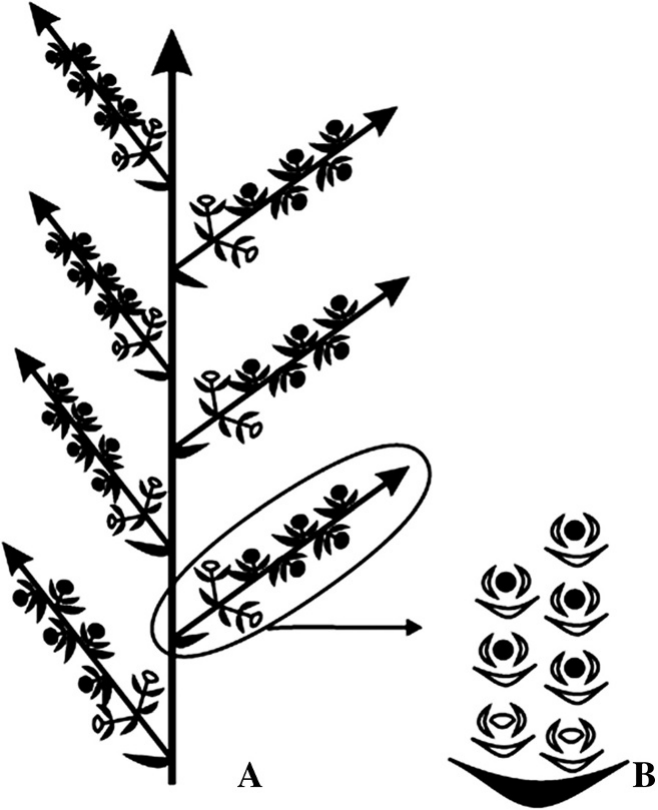
**Squematic representation of an inflorescence of** ***Vigna adenantha*** **. (A)** Scheme of the inflorescence. **(B)** Detail of a secondary axis. Semi-circles, flowers; circles, extrafloral nectaries.

**Figure 2:**
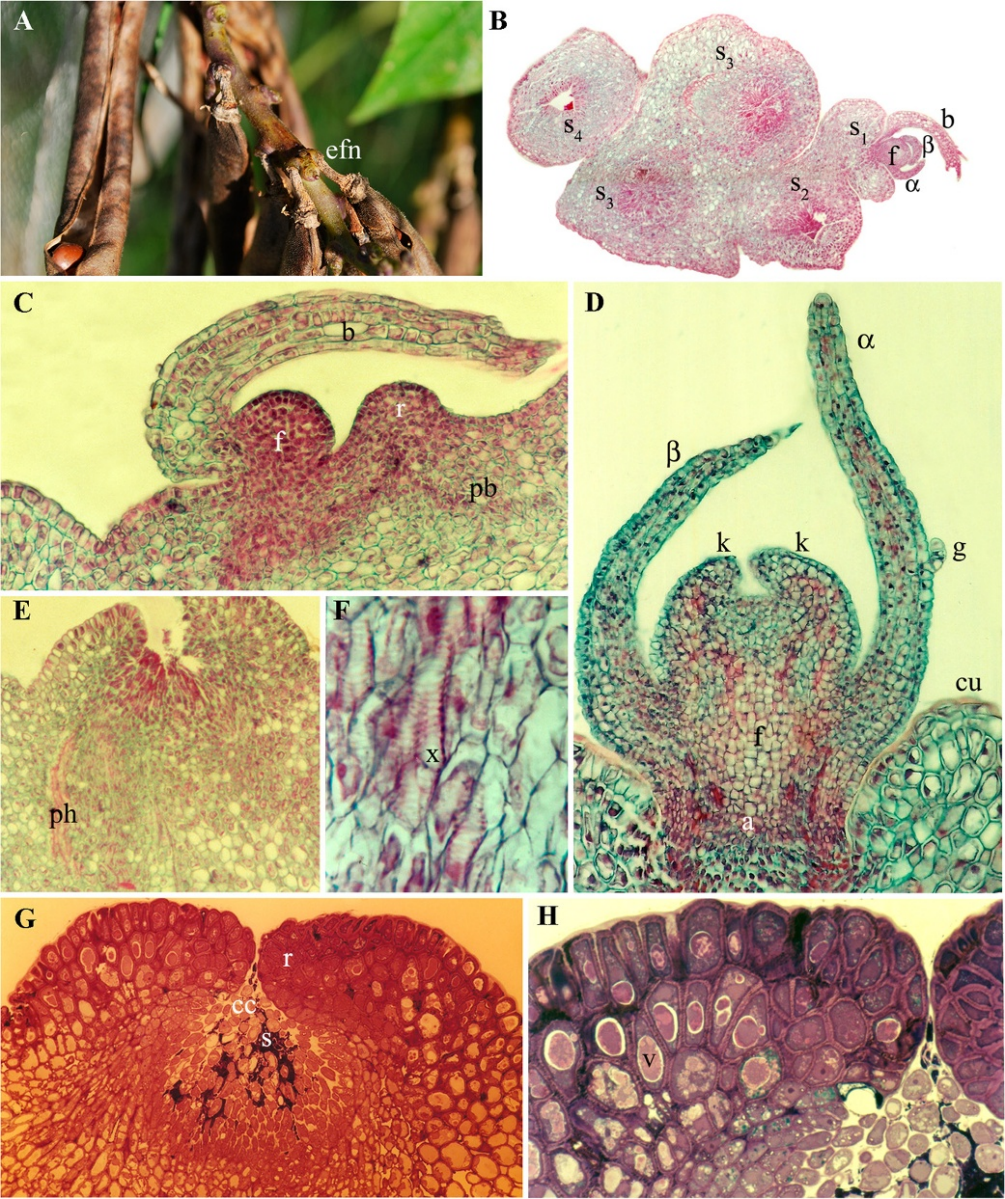
**Extrafloral nectary ontogeny in** ***V. adenantha*** **. (A)** View of an inflorescence globose secondary axis bearing 2 flowers that have set fruit and 1-7 extrafloral nectaries (EFNs). **(B)** Transverse section of an inflorescence secondary axis with a bud (f) covered by a tectrix bract (b) and bracteoles (α, β) and 4 nectaries in different stages (s_1_, stage 1; s_2_, stage 2; s_3_, stage 3; s_4_, stage 4). **(C-H)**: longitudinal sections of an EFN. **(C)** Stage 1: Tectrix bract (b) protecting a primordium, developing ring (r) and procambial bands (pb). **(D)** Stage 2: α and β bracteoles (α, β) with a glandular tricome (g), bud with two calix lobes (k), abscission zone (a), surrounded by the ring with cuticle (cu). **(E-F)** Section of the basal part of an EFN showing phloem cells (ph) and xylem cells (x). **(G)** Stage 3. The mature nectary stained with safranin-fast green, exhibiting the ring and the central cells (cc) with secretion (s) among them. **(H)** Stage 3. Detail of the ring stained with toluidine blue, with highly dense epidermal cells and parenchyma cells with vacuoles (v) filled with dense content. Scale bars: **(A)** = 7.5 mm, **(B-D)** = 4 μm, **(E, F)** =5 μm, **G** = 100 μm; **H** = 40 μm.

**Figure 3:**
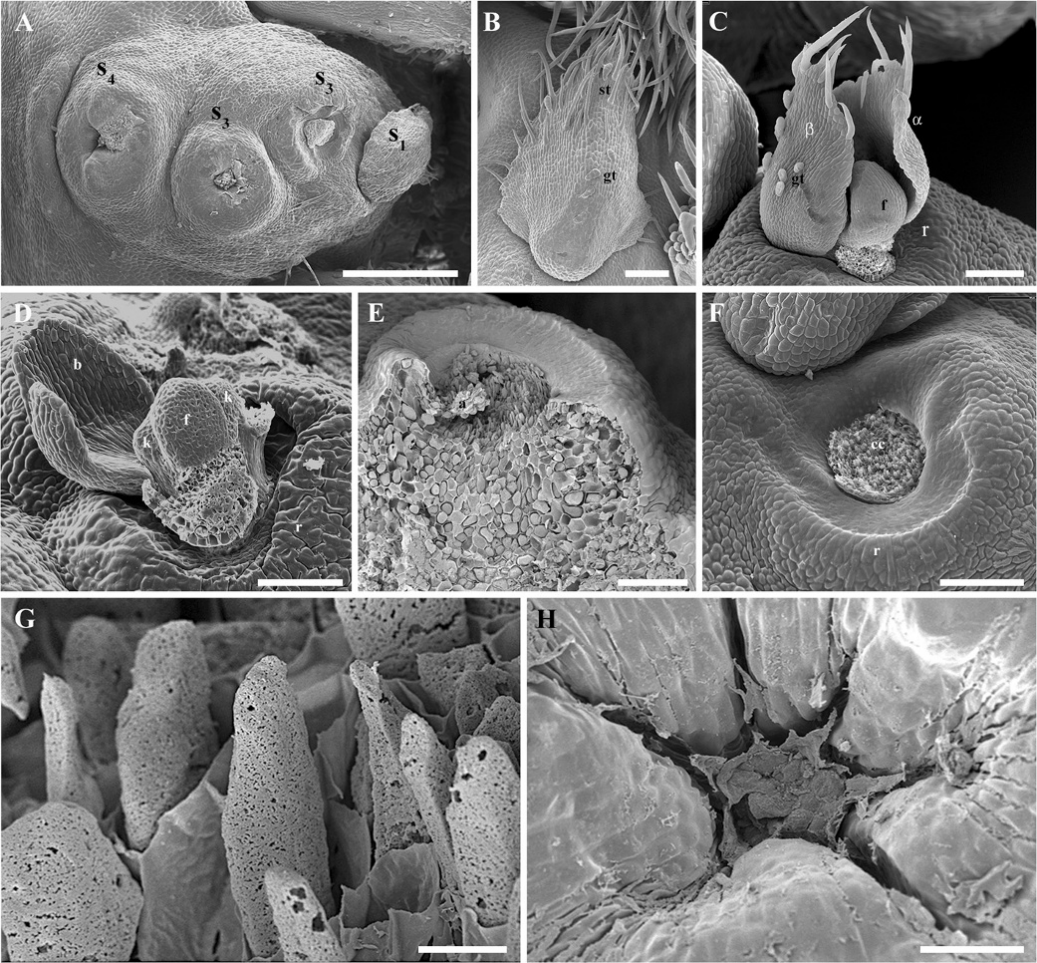
**SEM of the extrafloral nectary ontogeny of** ***V. adenantha*** **. (A)** globose secondary axis of the inflorescence with nectaries in different stage (s_1_, stage 1; s_3_, stage 3; s_4_, stage 4). **(B)** Stage 1. Tectrix bract with simple trichome (st) and glandular trichome (gt). **(C)** Stage 2. Scar of the tectrix bract α and β bracteoles (α, β) with simple and glandular trichomes, bud (f) and ring (r). **(D)** Stage 2. Scar of the tectrix bract, scar of the α bracteoles, β bracteole, bud with calix lobes (k) and ring. **(E)** Stage 3. Longitudinal section of an EFN with rests of the abscission zone (a). **(F)** Stage 3. Middle-moon shaped ring and the central cells (cc). **(G)** Stage 3. Detail of central cells with partially degraded walls. **(H)** Stage 4. Central cells completely desintegrated. Scale bars: **(A)** =500 μm; **(B)** =30 μm; **(C, D, F)** =100 μm; **(E)** =20 μm **(G)** =1 μm; **(H)** =10 μm.

The inflorescences of *Vigna adenantha* have 9 or more nodes in the main axis; a globose secondary axis originates in each node, which bears 2 flowers and 5 to 7 EFNs (Figure 1). In the first node of this secondary axis three buds originate, two of them develop into flowers, the third one becomes in an EFN, in the following nodes, only EFNs develop (Figure 2A, B). The nectaries are sessile, tightly disposed (Figure 3A). Each one has a structure that consists of a group of cells located in the center, surrounded by a complete or middle-moon shape ring (Figure 3A). The formation of a middle moon or complete ring depends on the relative position of each EFN in the secondary axis and the consequent available space.

### Ontogeny

Four stages of development were distinguished in the EFNs. In the following description the development of the first EFN is assessed and it is correlated with the microsporogenesis, microgametogenesis, megasporogenesis and megagametogenesis of the flowers of the same node. The following EFNs of the secondary axis have the same ontogeny.

Stage 1. The bud of the EFN is placed in the axil of a bract of the secondary axis and is constituted by a protodermis which protects the underlaying meristematic tissue (Figure 2C). The bract has simple and glandular pluricellular trichomes in its abaxial surface (Figure 3B). Around or beside the bud, a ring begins to develop; it is constituted by a protodermis which protects the underlaying parenchyma. Beneath the bud as well as beneath the developing ring, procambial bands can be observed which originate phloem and xylem (Figure 2C).

In the same node of the secondary axis, two floral buds are developing as floral primordia, the microspore mother cells in their first meiotic division are found in the anthers, but the megaspore mother cell has not differentiated yet in the ovules.

Stage 2. Beneath the bract, two bracteoles are formed (Figure 2D), which are pilose in their abaxial face, with simple and glandular pluricellular trichomes (Figure 3C). In the meristematic apex of the bud, sepals primordia can be observed (Figures 2D and 3D). At the base of the bud, a band of cells more vacuolated than the surrounding ones are distinguished, which would correspond to a future abscission zone (Figure 2D). Below this zone, there are longitudinally enlarged cells, with thin primary walls and large nuclei. The ring of the future EFN is already totally differentiated: it is constituted by an epidermis and a parenchyma without intercellular spaces, with highly vacuolated cells and with conspicuous nuclei (Figure 2D). At the base of the EFN in formation, vascular bands are observed (Figure 2E, F).

In the corresponding floral buds of the same node, microspore tetrads are present in the anthers and the megaspore mother cell begins the meiotic division in the ovule.

Stage 3. The bud of the EFN does not continue its development and detaches from the abscission zone, leaving exposed the longitudinally enlarged cells in the center of the nectary (Figures 2G and 3E, F). These central cells elongate longitudinally even more, remaining as papillae, whose walls partially degrade exhibiting perforations (Figure 3G). Bellow these central cells there is a parenchymatic tissue. The secretion, which stains strongly with safranin-fast green (Figure 2G) and toluidine blue (Figure 2H), accumulates among the central cells. The parenchyma cells of the ring have conspiscuous vacuoles with content that also stain intensely with toluidine blue (Figure 2H).

In the corresponding floral buds of the same node, the anthers have pollen grains already formed and some tapetum rests, while in the ovules the megaspore is in the mitotic process which ends in the megagametophyte formation.

Stage 4. Nectar secretion ceases and the central cells desintegrate (Figure 3H).

In the corresponding floral buds of the same node anthers are dehiscent and the tapetum is completely dissolved while in the ovules, the megagametophyte is totally formed. At this moment, anthesis begins.

### Ultrastructure of the secretory stage (stage 3)

#### Ring

**Figure 4:**
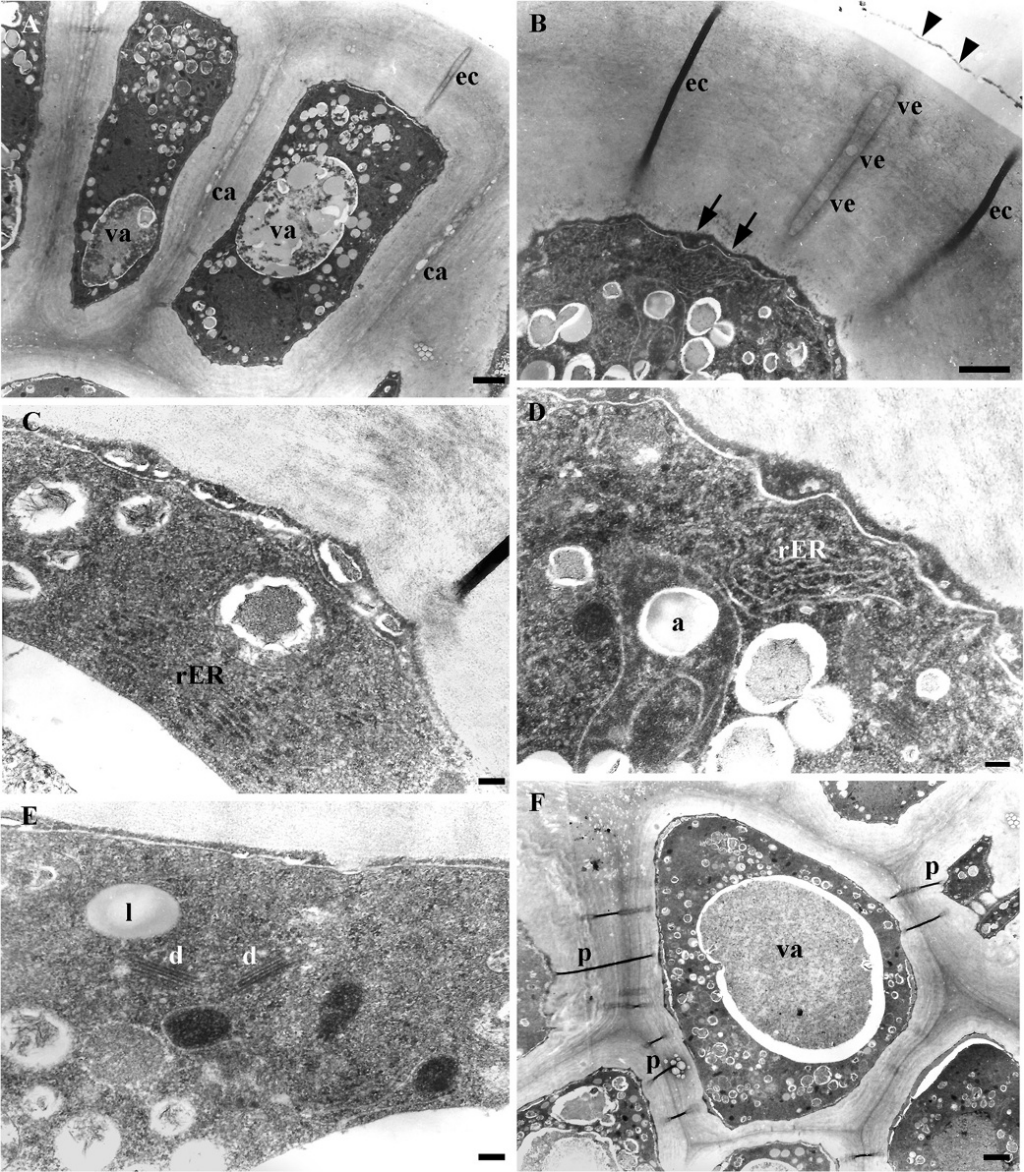
**TEM of the EFN in stage 3 of** ***V. adenantha*** **(ring). (A-B)** The epidermal cells. **(A)** General view of cell with vacuole (va), cavities in the middle lamellae (ca) and ectodesmata (ec). **(B)** Detail of the outer tangential wall with highly electrondense content outside the invaginated plasmalemma (arrows), inside the ectodesmata, which also have vesicles (ve), and outside the wall (arrowhead). **(C-F)** Parenchyma cells. **(C)** Detail of invaginated plasma membrane and rough endoplasmic reticulum (rER). **(D)** Rough endoplasmic reticulum and amyloplasts (a). **(E)** Dictyosomes (d), lipidic globules (l) and mitochondria (m). **(F)** Plasmodesmata (p) connecting adyacent cells and vacuole with fibrilar content (va). Scale bars: **(A, F)** =2 μm; **(B)** =1 μm; **(C-E)** =200 nm.

The epidermal cells of the ring exhibit a very thick outer tangential wall, crossed by conspicuous ectodesmata (Figure 4A), some of them filled with a highly electrondense content or with vesicles inside (Figure 4B). An equivalent electrodense content accumulates outside the invaginated plasmalemma (Figure 4B, arrows) and outside the outer tangential wall (Figure 4B, arrowheads). The middle lamella of the radial walls appears distended and cavities can be seen along it (Figure 4A). The parenchyma cells have very dense cytoplasm, evident vacuoles with fibrilar content of varying electrondensity, rough endoplasmic reticulum, numerous free ribosomes, dictyosomes, abundant amyloplasts with one to several starch granules, mitochondria and lipidic globules (Figure 4, C-F). Plasmodesmata can be observed between parenchymatic cells (Figure 4F).

#### Central cells

**Figure 5:**
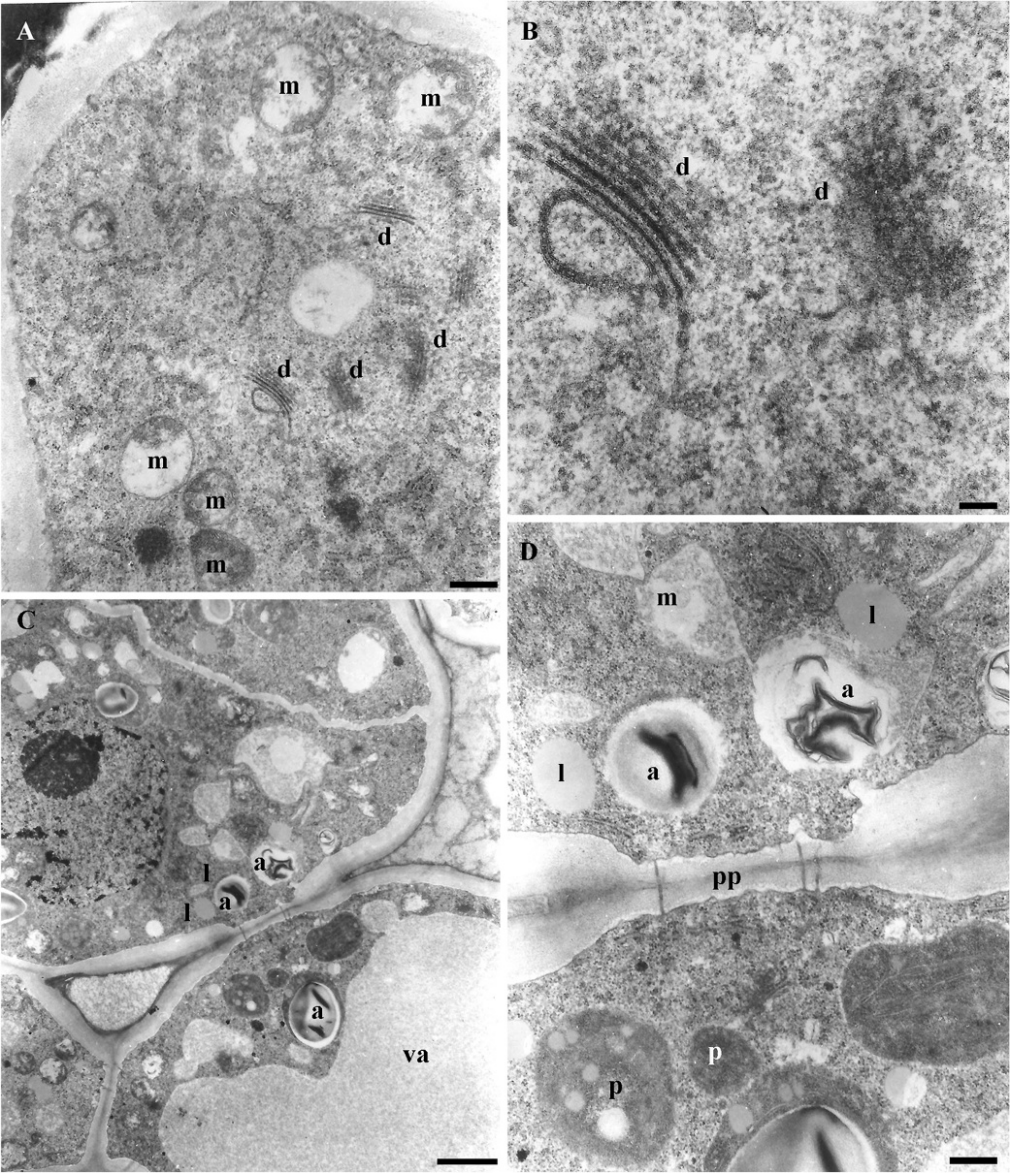
**TEM of the EFN in stage 3 of** ***V. adenantha*** **(central cells). (A-B)** Papillose cells. **(A)** Dictyosomes (d) and mitochondria (m). **(B)** Detail of **(A)**. Dyctiosomes and free ribosomes. **(C-D)** Underlying parenchyma cells. **(C)** Amyloplasts (a), vacuole with medium electrondense content (va), lipidic globules (l). **(D)** Detail of **(C)**. Amyloplasts, lipidic globules, plastids (p), mitochondria and plasmodesmata (pp). Scale bars: **A**, **D** = 0,5 μm; **B** = 0,1 μm; **C** = 2 μm.

The cytoplasm of the elongated and papillose cells has numerous free ribosomes, abundant mitochondria and dictyosomes (Figure 5A, B). The underlying parenchymatic cells exhibit vacuoles with medium-electrondense content, amyloplasts, plastids that contain both starch granules and lipidic globules, free lipidic globules and mitochondria (Figure 5 C, D). Plasmodesmata connect these cells (Figure 5D).

### Nectary activity period

During the inflorescence development, once the first EFN of a secondary axis arrives to stage 3, the second EFN begins to differentiate in the next node and so on, in the acropetal sense of the secondary axis (Figures 2B and 3A).

When the fruits have seeds with embryos in the globular stage, at least one EFN in stage 4, one EFN in stage 3 and one EFN in stage 1 coexist in the same secondary axis of the inflorescence, so the EFNs activity is continuous during maturation of the fruits (Figure 2A).

### Nectar concentration

Nectar removed from nectaries in stage 3 was highly viscose, the concentration ranged from 0.02 to 12.5 (3.01 ± 4.78, n = 5) percent of total sugars. During sunny days with low ambient humidity, crystallization of the nectar was observed on the EFN surface.

## Discussion

The EFN of *V. adenantha* originates from the abscission of a floral bud that interrupts its development. This cessation of meristematic activity and detachment of the developing flower was also observed in *Macroptilium* species (Díaz-Castelazo et al. [2005]) and the origin of EFNs from aborted flower buds in inflorescences has been suggested for other Legumes (Ojehomon [1968]; Tucker [2003]).

This kind of nectaries would fit the “Hochnektarien” (elevated nectaries) type according to the classification proposed by Zimmerman ([1932]) and accepted by Elias ([1983]), since they are elevated from the surrounding tissues.

Ojehomon ([1968]) analysed the development of the glands in *Vigna unguiculata* and described them as “cushion units”, inferring that the secretion is the result of excretion of cellular products, and discarding their function as EFNs. The present results agree with the observations on the ontogeny described by Ojehomon ([1968]) but not with the role that was suggested.

Kuo and Pate ([1985]) described the EFNs in the secondary axis of the inflorescences of *Vigna unguiculata* as a compound structure formed by various conical secretory subunits but they did not clarify if all of them are active at the same time. They observed that each cone was formed by secretory parenchyma whose production (nectar plus cellular debris) was discharged through an orifice surrounded by a ring constituted of epidermis and parenchyma. In *V. adenantha* the EFNs are not compound structures because every EFN of each secondary axis originates independently and reaches the secretory stage at a different moment, following an acropetal sequence, because they are developing floral buds that abort subsequently. According to this, from the initiation of the floral buds that will produce flowers, there is always a nectary in active secretion in the same node until the fruit ripening of those flowers (stage 3). In other Legumes, the extrafloral nectaries are also active until pod maduration (Pate *et al.*[1985]; CAB [2004]).

Kuo and Pate ([1985]) pointed out that the vascular supply of the EFNs of *V. unguiculata* is only constituted by phloem; contrarily, in *V. adenantha* the vascular supply is constituted by phloem and xylem.

Kuo and Pate ([1985]) did not mention that the cells of the ring secrete, in contrast to the observations in this work. Those authors postulated that the secretion would occur through the intercellular spaces underlying the central cells. In *V. adenantha*, all the cells (the central ones and the ones from the ring) secrete.

Nectar would be liberated by three ways: through the ectodesmata of the epidermal cells of the ring (with previous accumulation outside the plasma membrane), through the cavities of the middle lamellae present between these cells and through the partially degraded walls of the central cells. The ectodesmata and the cavities would aid the secretion crossing the thick outer tangential epidermal cell walls of the ring. A thin cuticle is usually present on the nectary epidermis, which becomes thinner as nectar secretion proceeds (Nepi [2007]). Cavities have been observed inside thick tangential walls of secretory cells (Aliscioni et al. [2009] and cites therein).

The presence and abundance of dictyosomes and endoplasmic reticulum in the ring and in the central cells, as well as the vesicles along the ectodesmata, suggest granulocrine secretion, unlike the conclusions by Kuo and Pate ([1985]), who interpreted that nectar would follow an apoplastic route and flow through the orifices of the conical subunits.

The scant measurements of extrafloral nectar concentration involving several plant families and different EFN types range from very dilute (0.73) to considerable concentrated (80) total sugar percent even within the same nectary type (see Díaz-Castelazo et al. [2005]), although ants, the most frequent visitors of these glands, prefer concentrated nectar, i.e., 40-50 total sugars % (Nicolson [2007]). The high variation of extrafloral nectar concentration has been attributed to the influence of microenvironment conditions surrounding the usually exposed EFNs (Koptur [1992]), in contrast to the more concealed floral ones whose concentration does not vary as much.

## Conclusions

The EFNs in *V. adenantha* are generated from aborted floral primordia. The secretion is granulocrine. In each secondary axis of the inflorescence, several EFNs originate and secrete successively and independently until fruit ripening; this succession provides constant reward to patrolling ants, which would protect the developing fruits until maturation.

This is the first report in which the development of the EFNs is correlated with the development of the flowers of the same node.

## Authors’ original submitted files for images

Below are the links to the authors’ original submitted files for images. Authors’ original file for figure 1 Authors’ original file for figure 2 Authors’ original file for figure 3 Authors’ original file for figure 4 Authors’ original file for figure 5

## Abbreviations

BAFC: Herbario de la Facultad de Ciencias Exactas y Naturales (Universidad de Buenos Aires)
EFNs: Extrafloral nectaries
FAA: Formaldehide, alcohol, acetic acid, water
OM: Optical microscopy
OsO_4_: Osmium tetroxide
SEM: Scanning electronic microscopy
TEM: Transmission electron microscopy
