# Supplementary figures and images for: Ontogeny of the extrafloral nectaries of Vigna adenantha (Leguminosae, Phaseolae) and its relation with floral development
Source: Bot Stud. 2014 Dec 29;55:74. doi: 10.1186/s40529-014-0074-2 (PMC5430357; doi:10.1186/s40529-014-0074-2)

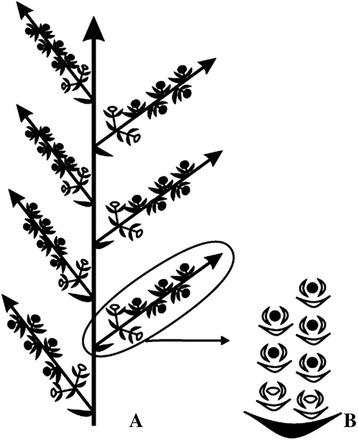

Supplement: Supplementary file 1 — Authors’ original file for figure 1 [file 40529_2014_9074_MOESM1_ESM.gif]

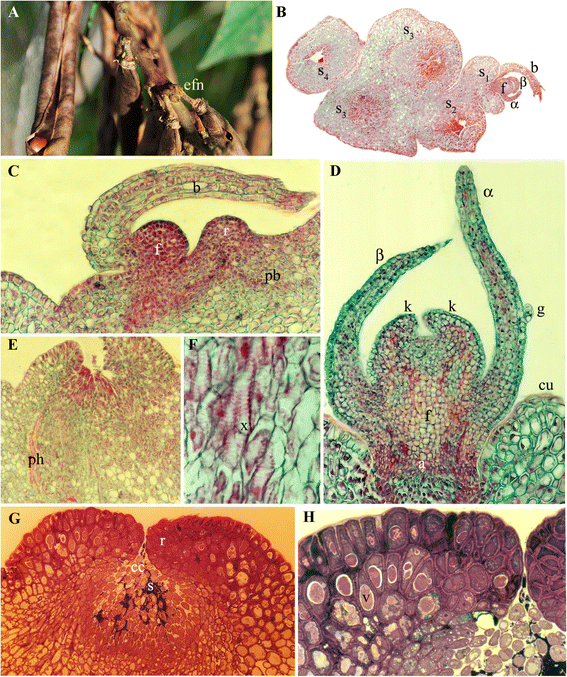

Supplement: Supplementary file 2 — Authors’ original file for figure 2 [file 40529_2014_9074_MOESM2_ESM.gif]

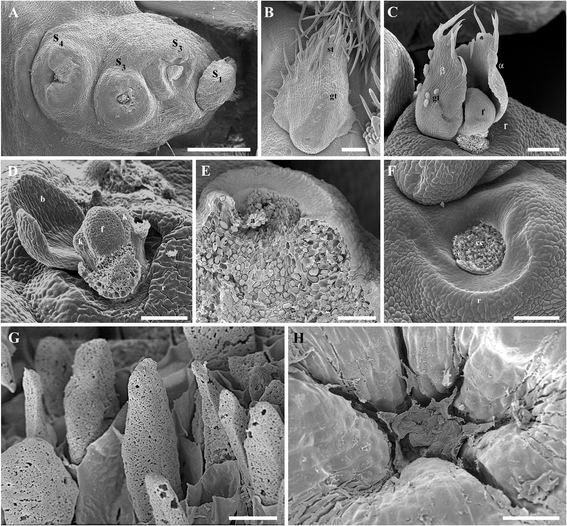

Supplement: Supplementary file 3 — Authors’ original file for figure 3 [file 40529_2014_9074_MOESM3_ESM.gif]

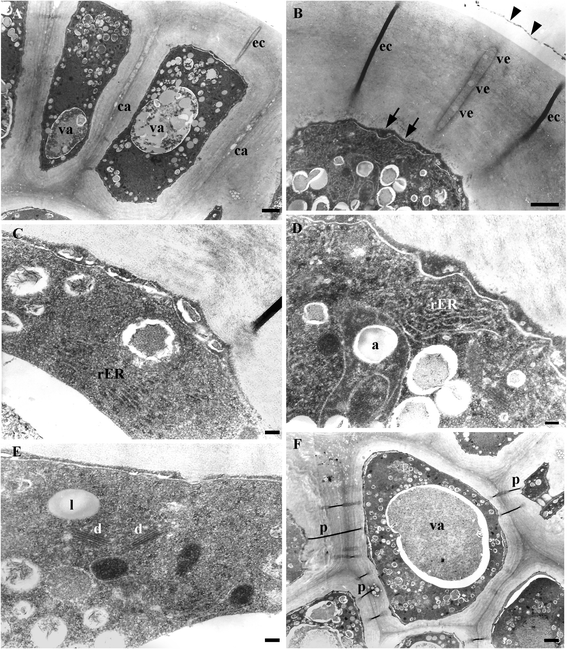

Supplement: Supplementary file 4 — Authors’ original file for figure 4 [file 40529_2014_9074_MOESM4_ESM.gif]

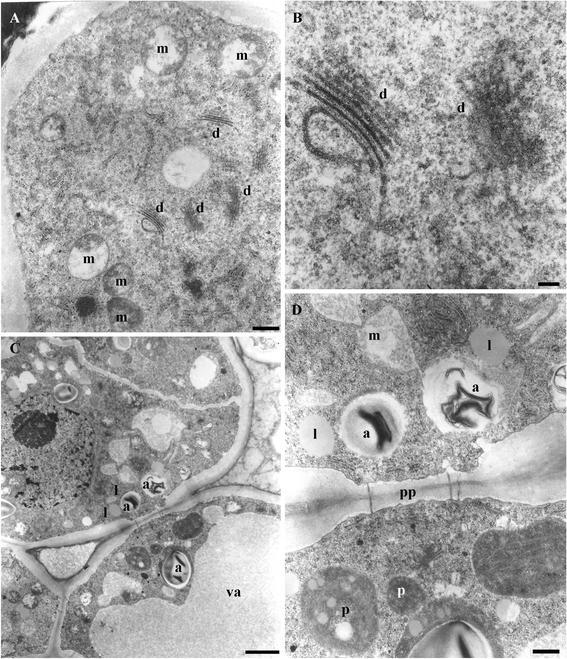

Supplement: Supplementary file 5 — Authors’ original file for figure 5 [file 40529_2014_9074_MOESM5_ESM.gif]
